# Evaluating the feasibility and predictive accuracy of biodynamic imaging to platinum-based chemotherapy response in esophageal adenocarcinoma

**DOI:** 10.3389/fonc.2024.1429343

**Published:** 2024-09-30

**Authors:** Ali Ajrouch, Ben Krempley, Ahmad Karkash, John M. Dewitt, Mohammad Al-Haddad, Dawith Lim, David Nolte, John Turek, Susan M. Perkins, Shadia I. Jalal

**Affiliations:** ^1^ Division of Hematology Oncology, Indiana University Melvin and Bren Simon Comprehensive Cancer Center, Indianapolis, IN, United States; ^2^ Division of Gastroenterology and Hepatology, Indiana University School of Medicine, Indianapolis, IN, United States; ^3^ Department of Physics, Purdue University, West Lafayette, IN, United States; ^4^ Department of Biostatistics and Health Data Science, Indiana University School of Medicine, Indianapolis, IN, United States

**Keywords:** esophageal adenocarcinoma, biodynamic imaging, chemotherapy response prediction, patient-specific modeling, digital holography, precision oncology, platin agents

## Abstract

**Background:**

Esophageal cancer management lacks reliable response predictors to chemotherapy. In this study we evaluated the feasibility and accuracy of Biodynamic Imaging (BDI), a technology that employs digital holography as a rapid predictor of chemotherapy sensitivity in locoregional esophageal adenocarcinoma.

**Methods:**

Pre-treatment endoscopic pinch biopsies were collected from patients with esophageal adenocarcinoma during standard staging procedures. BDI analyzed the tumor samples and assessed *in vitro* chemotherapy sensitivity. BDI sensitivity predictions were compared to patients’ pathological responses, the gold standard for determining clinical response, in the surgically treated subset (n=18).

**Result:**

BDI was feasible with timely tissue acquisition, collection, and processing in all 30 enrolled patients and successful BDI analysis in 28/29 (96%) eligible. BDI accurately predicted chemotherapy response in 13/18 (72.2%) patients using a classifier for complete, marked, and partial/no-response. BDI technology had 100% negative predictive value for complete pathological response hence identifying patients unlikely to respond to treatment.

**Conclusion:**

BDI technology can potentially predict patients’ response to platinum chemotherapy. Additionally, this technology represents a promising step towards optimizing treatment strategies for esophageal adenocarcinoma patients by pre-emptively identifying non-responders to conventional platinum-based chemotherapy.

## Introduction

Esophageal adenocarcinoma (EA) is a significant health concern worldwide. In the United States, it accounts for over 16,000 deaths annually (1). Most patients are diagnosed with locally advanced stage II or III disease, for which the gold standard treatment is preoperative CRT (CRT) followed by surgery in eligible patients. Recently, adjuvant immunotherapy was integrated into the treatment of those with residual disease at the time of surgery (2, 3). Nonetheless, the 5-year survival rate for locoregional EA remains below 20% (4).

Neoadjuvant CRT provides an absolute benefit of 13% improvement in 5-year survival for locally advanced esophageal cancer compared to surgery alone (5, 6). The benefits of neoadjuvant chemotherapy and radiation are most pronounced among patients with an excellent histopathological response. However, only 23% of patients with EA display a complete pathological response, as demonstrated by the CROSS trial (2). Conversely, patients with a poor response to CRT are more likely to have a poor prognosis and suffer from treatment-related toxicities with limited benefits. Studies estimate that 40% of patients treated with CRT suffer from toxicities while deriving limited benefit (2, 7–12). These toxicities range from financial and emotional burdens to physical side effects, such as fatigue, esophagitis, and bone marrow suppression.

The ability to accurately predict chemotherapy response would optimize treatment outcomes by identifying patients most likely to benefit from neoadjuvant therapy. It would also support likely non-responders by providing them with additional data for a more informed discussion regarding their prognosis and spare them unnecessary toxicities. Although several “Predictive Biomarkers” have been evaluated, none have been validated for predicting the response to platinum chemotherapy or taxanes, the standard EA chemotherapies. Additionally, the reliability of traditional staging imaging modalities, such as positron emission tomography (PET), in predicting treatment response remains uncertain (13).

We investigate Biodynamic Imaging (BDI) as a potential solution. BDI uses low-intensity light illumination to construct three-dimensional holographic reconstructions from depths up to one millimeter inside the tissue. These reconstructions allow it to capture and analyze intracellular motions in living tissue and cancer biopsies. Intracellular motions within the tissue produce signals that were shown to be modulated by tumor therapeutic agents in the laboratory setting. Capturing and interpreting these signals enables BDI to measure cellular responses to applied therapeutics (14–29)Preliminary laboratory BDI testing in ovarian cancer cell lines with different cisplatin sensitivities, in human epithelial ovarian cancer (30) and in canine multicentric lymphoma to predict doxorubicin sensitivity (31) supported its potential as a chemotherapeutic response predictor and pave the way for human trials.

In this phase 2 trial, we explore implementing BDI technology in a clinical setting and its potential predictive ability for chemotherapy response in locoregional EA by training a three-class neural network classifier for EA clinical chemotherapy response. The primary objective was to examine the feasibility of implementing the BDI technique in a clinical setting with respect to patient enrollment, tumor tissue acquisition, sample transport within 24 hours, sample processing, and the successful application of BDI technology. The secondary objective was to determine the correlation between the obtained BDI prediction and patients’ clinical response to chemotherapy, defined as the pathological response in the subset of patients who underwent surgery.

## Methods

### Eligibility criteria

We enrolled patients aged ≥ 18 years with untreated, nonmetastatic, histologically confirmed EA who were medically fit and willing to undergo chemoradiotherapy. The Institutional Review Board provided ethical approval, and all participants provided written informed consent.

### Specimen collection

Participants underwent endoscopic ultrasound (EUS) for standard disease staging, during which we collected a tumor pinch biopsy, the only sample collected for research. The tumor samples were sent to the laboratory for analysis, as described below. Subsequently, the participants received standard preoperative chemoradiotherapy followed by surgery if medically appropriate. Treatment and management decisions, including chemotherapy regimen selection, rested on the treating physician’s discretion and followed the standard of care. These decisions were made independently of the BDI results. The two main concurrent chemotherapy regimens were carboplatin with paclitaxel or cisplatin with 5-fluorouracil. We recorded the administered regimen to each patient and matched it to its representative BDI analysis arm. After completing CRT therapy, if patients underwent esophagectomy, we recorded their pathological response and evaluated the correlation between it and BDI’s response prediction. **Table 1** presents the criteria for assessing tumor pathological responses. We limited the correlation with BDI to the pathological results in the subset of patients who underwent esophagectomies, as surgery is the gold standard for determining pathological responses in locoregional EA. Pathological responses post CRT have been shown to correlate with overall survival in esophageal cancer (32). EUS post-chemoradiotherapy has limited specificity and sensitivity, and EUS biopsies post-chemoradiotherapy are limited by sampling errors (33–35). In addition, inflammatory changes on PET/CT limit the interpretation and determination of treatment response using this modality (36). BDI results from patients who did not undergo surgical resection were classified using the neural network classifier but were not used for training or for calculating prediction accuracy.

**Table 1:** Criteria for assessing tumor pathological response (modified from the College of American Pathologists guidelines).

Response	Definition
**No residual tumor/Complete Response**	Grade 0: no viable cancer cell, 0% tumor.
**Near Complete/Marked Response**	Grade 1: single/rare groups of cancer cells, 0 - <10% residual tumor.
**Moderate/Partial Response**	Grade 2: residual cancer with regression, 10-50% residual tumor.
**Poor/No definite response**	Grade 3: No tumor regression, >50% residual tumor.

**Figure 1:**
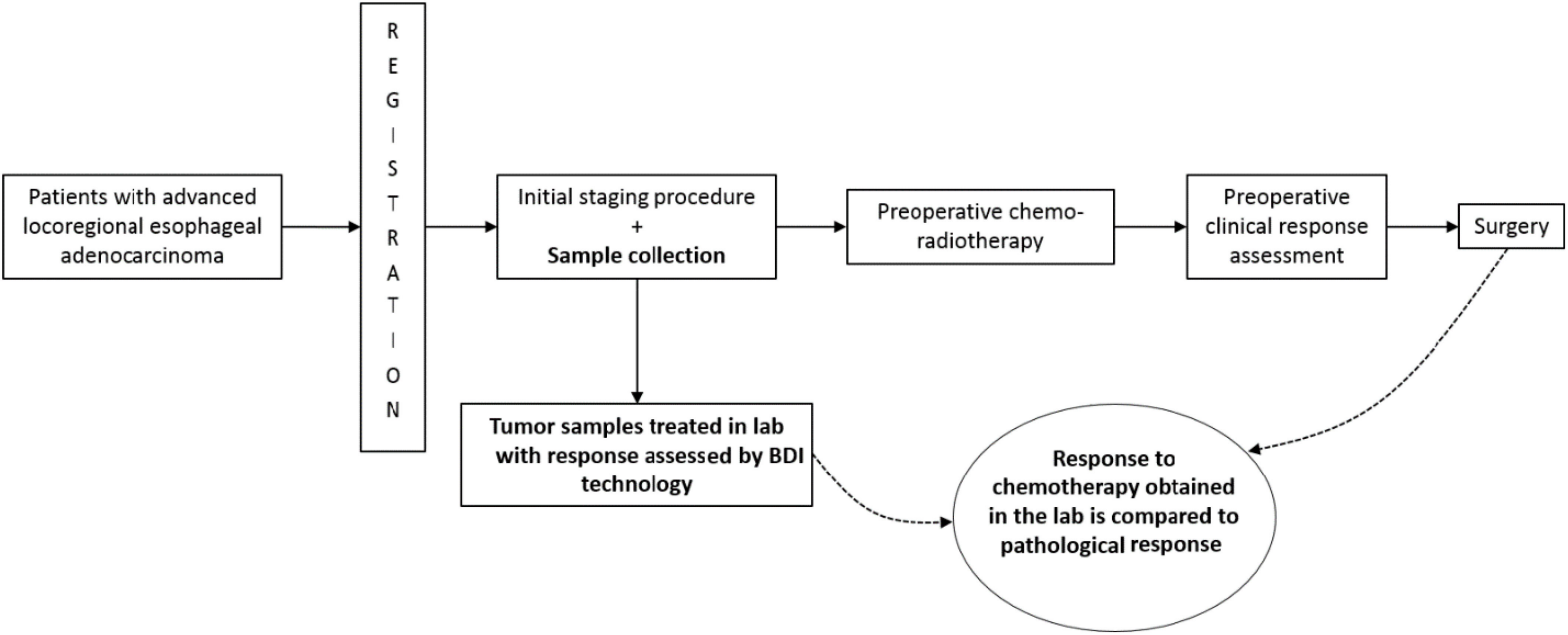
Outline of the study design. Bolded areas represent study interventions.

Radiation effects were not modeled *in vitro* as there is no simple way to mimic radiotherapy in the laboratory setting. We accept this as a potential limitation of the present study. Any adverse events related to the study procedure were recorded and followed up. All treating specialists were blinded to BDI prediction. **Figure 1** outlines the study’s design.

### Biopsy sample processing

Tumor biopsy samples used for biodynamic imaging were chilled (4°C) the same day and dissected into multiple pieces of one mm^3^. Between 16 and 32 pieces per patient were immobilized in 96-well plates using agarose and immersed in RPMI 1640 growth medium. The wellplate with the biopsy samples was then mounted onto the sample stage of the BDI system for data acquisition. After acquisition of a 4-hour baseline, samples were treated with cisplatin, carboplatin, 5-fluorouracil, and paclitaxel individually and with each combination of (cisplatin + 5-fluorouracil) and (carboplatin + paclitaxel). The four chemotherapy agents were selected based on their prevalent use in treating EA and their varied action mechanisms, representing the therapeutic strategies employed in current clinical practice. The dynamic spectra were acquired over 10 hours after the application of the treatment *in vitro*, capturing the early physiological responses of the samples to the treatments likely related to the uptake facility of small-molecule drugs by living cells within the biopsy. The biopsy samples have been shown to fully maintain their health during this period. Afterward, the culture medium was replaced with 10% neutral buffered formalin for preservation and stored at 4°C.

### Biodynamic imaging

Biodynamic Imaging (BDI) is a dynamic-contrast, holographic optical coherence tomography technique. The optical principle behind the technique is off-axis holography; the interference of the object and reference fields with a small offset angle creates a hologram on the Fourier plane of the digital camera, and a numerical spatial transform of the hologram yields a reconstruction of the object field on the image plane. The offset between the two beams creates spatial separation of the reconstructed components due to the spatial carrier wave associated with the interference fringe pattern. BDI is optimized to have high sensitivity to small-scale intracellular motions. These motions are captured by the dynamic speckle pattern on the hologram, which in turn is captured by the intensity fluctuations of the reconstructed field which can be used as a surrogate observable to examine the dynamics of the living tissue sample.

**Figure 2:**
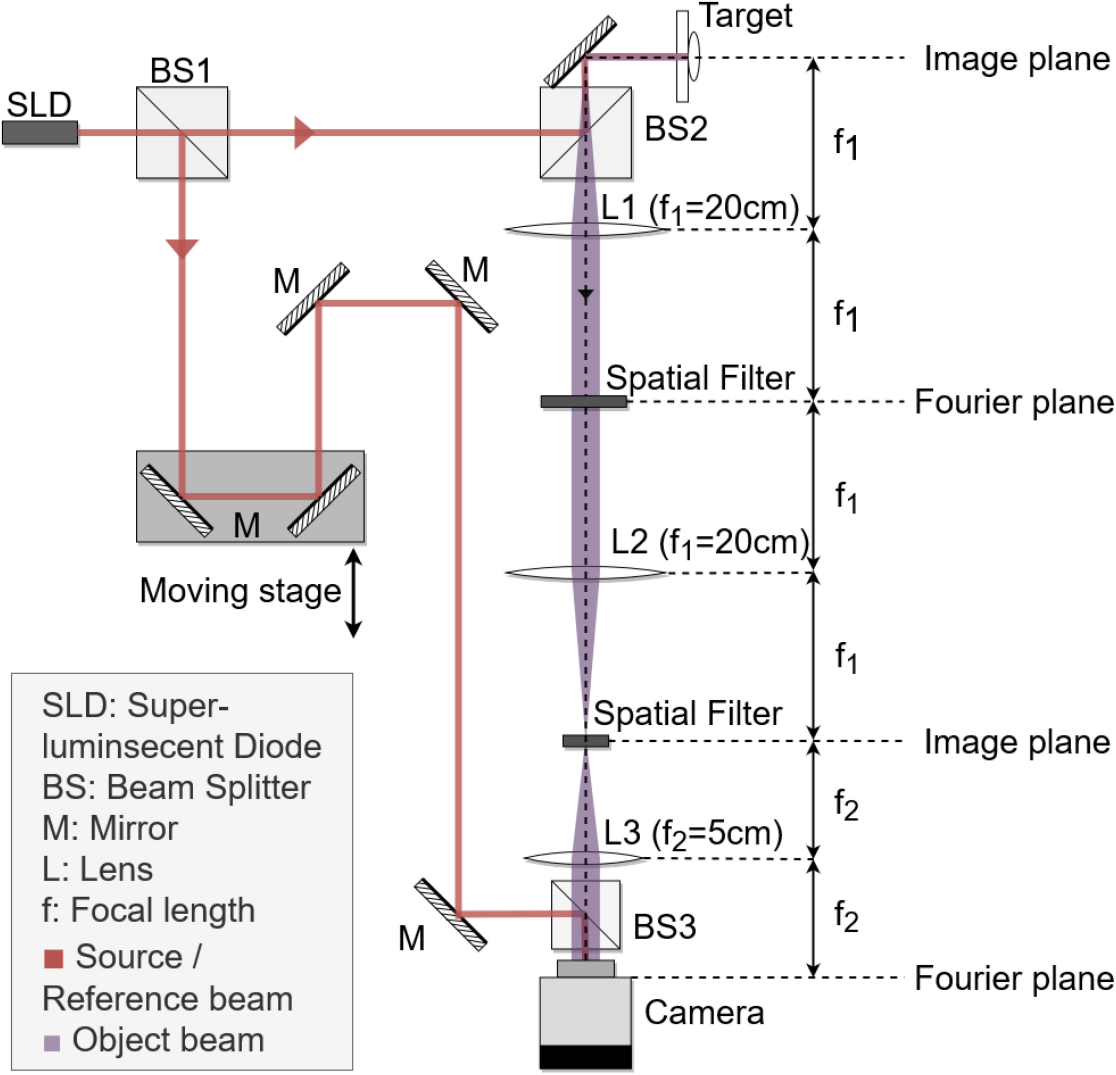
A schematic diagram of the Mach-Zehnder interferometer used to perform biodynamic imaging (BDI). The reference arm contains a 180° reflector mounted on a motorized stage to control the coherence depth for the optical sectioning. The object arm contains a series of optical Fourier transforms with spatial filters (apertures). The object and reference beams are recombined with a slight angle offset at the beam splitter (BS3) and projected onto the camera located on the Fourier plane, where the interferogram is recorded.

For this study, a Mach-Zehnder interferometer with short-coherence superluminescent diode (SLD) source of λ = 840nm was used to implement the BDI technique. A schematic of the optical system is shown in **Figure 2**. The low-coherence light and variable optical path length (OPL) on the reference arm enable coherence gating, where photons with matching OPL are coherent with respect to the reference and form a stable interference pattern, while photons whose OPL stretches outside the coherence length contribute only to random noise. Coherence gating allows a consistent acquisition of thin optical sections (~20 μm) of the sample.

### BDI features

**Figure 3:**
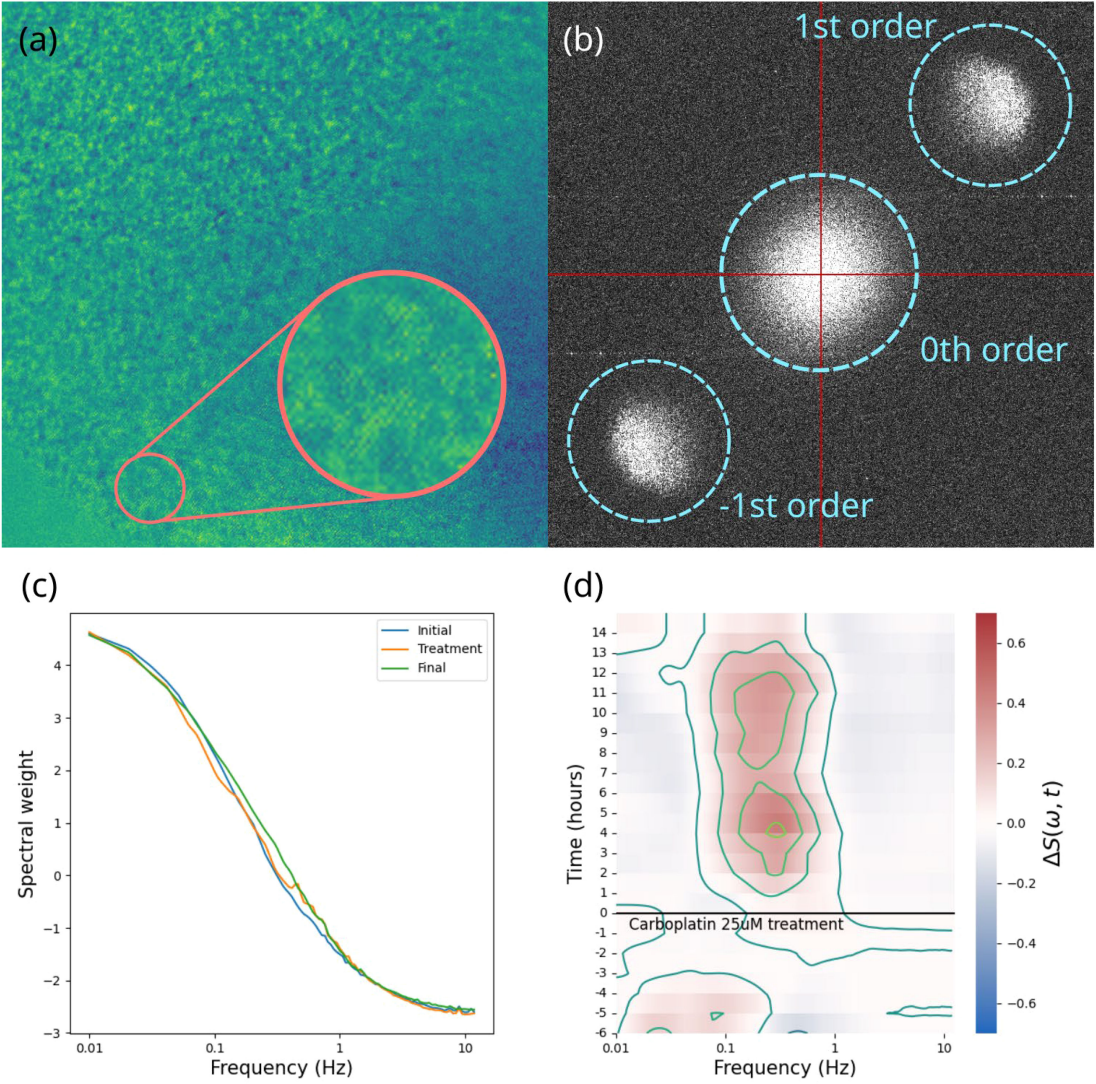
A visual summary of the data processing steps involved in biodynamic imaging. **(A)** the raw hologram captured by the camera, showing the interference fringes and speckle pattern; **(B)** 2D digital Fourier transform of the hologram showing the zeroth order terms (spatial autocorrelation) in the center, the object field and its phase conjugate offset from the center; **(C)** an averaged power spectrum; **(D)** differential spectrogram showing the change in power spectral weights over the course of the experiment for one sample.

BDI data consisting of high-frame-rate dynamic speckle images were converted into fluctuation power spectra averaged over all the sample pixels. Each power spectrum corresponds to an approximately 40-minute time frame. Several pre-treatment time frames established each sample’s baseline, while post-treatment frames captured drug-induced changes. Data aggregated from multiple time frames for each sample were converted into a differential spectrogram, i.e., a time-frequency representation of the relative change in spectral power, offering the “fingerprint” of the drug’s action on that sample (37). A visual summary of the data processing steps is shown in **Figure 3A** comprehensive report on the process and specifications of the BDI technology and other tools used in this project are published in Hua et al. (2024) (38).

We analyzed these fingerprints using time-frequency masks to isolate spectrogram sections previously correlated with biological function. For instance, concurrent high and low-frequency enhancements correlate with apoptosis. Hence, this concurrence is used as a biomarker. Various biomarkers were assessed, including integrated post-treatment power over the entire spectrogram, selected frequency bands, strong inhibition occurrence, overall spectra frequency shift, and collective fluctuation amplitude. We utilized the strongest correlating biomarkers exclusively to generate tumor cell survival predictive classifications presented as Complete Response (Grade 0), Marked Response (Grade 1), and Partial/No Response (Grade 2 or 3). Of these, the dominant treatments that best differentiated the responsive from the non-responsive patients were cisplatin, paclitaxel and the combination cisplatin+5fu. The dominant biomarkers were broad-frequency inhibition, change in metabolic activity, and shift in intracellular speeds.

### Neural network with triplet loss

The biomarkers were narrowed down to 20, with the strongest clinical outcome correlations. These were used as the input to a minimal two-hidden-layer neural network (NN) model with 20 neurons in the input layer, 20 in the first hidden layer, 10 in the second hidden layer, and 3 in the output layer. The NN was trained with the Adam update Matlab algorithm on a triplet loss function (39) for dimensionality reduction from D = 20 to D = 3. The three-dimensional output of the neural net was the input to a k-means clustering algorithm to generate the three-class classifier. The classifier was trained and validated using a one-left-out (OLO) approach in which each successive patient was held out of the training and then classified by the network trained on the remaining patients. Only patients with clinical pathology outcomes were used for training. Each patient receives a likelihood of belonging to each class. A table presenting BDI predicted response to chemotherapy and clinical pathological response to chemotherapy after surgery by subject is available in Appendix A.

### Statistical methods

Given the exploratory nature of this pilot study, our analysis is primarily descriptive, focusing on the feasibility of the BDI application and its predictive accuracy, which was limited to patients with available pathological responses. In a preclinical trial of biodynamic imaging for canine non-Hodgkin’s lymphoma sensitivity to doxorubicin with 10 dogs, the assay had a 90% correct classification rate (1 false case out of 10). Of the 6 dogs that were clinical responders, 5 of 6 were classified as responders by BDI. Of the 4 dogs that were clinical non-responders, all four were classified as non-responders by BDI. If our true correct classification rate in this study is 90%, with a sample size of n=15, a 95% two-sided confidence interval around that percentage would cover between 75% and 100%. However, because some samples may have quality problems (i.e., too little material, too heterogeneous material), up to 15 additional subjects were planned to be enrolled (n=30) to ensure 15 samples suitable for analysis, yielding an adequate estimate of the correct classification rate at this stage of the research. A point estimate and a 95% exact confidence interval were calculated. **Table 2** lists the pathological response and the equivalent BDI prediction.

**Table 2:** Clinical pathological response and equivalent BDI clinical prediction.

Path Response	BDI Response
**Grade 0**	Complete Response (Sensitive)
**Grade 1**	Marked Response (Mixed)
**Grade 2 or 3**	No Response (Resistant)

## Results

### Biodynamic imaging is feasible in the clinical setting

We enrolled 30 patients, later withdrawing one found to have metastatic disease, resulting in 29 subjects eligible for BDI analysis. Among the 29 patients, 18 proceeded to surgery and had their pathological responses documented. Among them, 6 patients achieved a grade 0 pathological response, 6 patients had grade 1 response, 4 patients had grade 2 response, and 2 patients had grade 3 response. The remaining 11 (37.93%) underwent chemoradiotherapy without surgery for several reasons, including poor recovery from chemoradiotherapy, patient choice, or surgeon’s preference. **Table 3** summarizes the demographics and clinical characteristics of our complete cohort and the surgery subset.

**Table 3:** Demographics and baseline clinical characteristics of all participants and those receiving surgery.

Characteristic	Enrolled Subjects (n=30)	Surgery Recipients (n=18)
**Age at Enrollment, mean (SD)**	66.0 (13.2)	60.9 (10.3)
Sex		
** Female, n(%)**	6 (20.0)	2 (11.1)
** Male, n(%)**	24 (80.0)	16 (88.9)
Race		
** White, n(%)**	29 (96.7)	17 (94.4)
** Unknown, n(%)**	1 (3.3)	1 (5.6)
Ethnicity		
** Non-Hispanic, n(%)**	27 (90.0)	16 (88.9)
** Unknown, n(%)**	3 (10.0)	2 (11.1)
Disease Stage at Specimen Collection		
** IB, n(%)**	4 (13.3)	2 (11.1)
** IIB, n(%)**	5 (16.7)	4 (22.2)
** IIA, n(%)**	1 (3.3)	0 (00.0)
** IIIA, n(%)**	13 (43.3)	8 (44.4)
** IIIB, n(%)**	2 (6.7)	1 (5.6)
** IIIC, n(%)**	1 (3.3)	0 (00.0)
** Unknown, n(%)**	3 (10.0)	2 (11.1)
** Missing, n(%)**	1 (3.3)	1 (5.6)
TMN Stage at Specimen Collection		
**T1N2M0, n(%)**	1 (3.3)	0 (00.0)
**T2N0M0, n(%)**	3 (10.0)	0 (00.0)
**T2N0MX, n(%)**	2 (6.7)	2 (11.1)
**T2N1M0, n(%)**	3 (10.0)	3 (16.7)
**T2N2MX, n(%)**	1 (3.3)	0 (00.0)
**T3N0M0, n(%)**	2 (6.7)	1 (5.6)
**T3N0 MX, n(%)**	1 (3.3)	1 (5.6)
**T3N1 M0, n(%)**	8 (26.7)	1 (5.6)
**T3N1MX, n(%)**	3 (10.0)	5 (27.8)
**T3 N2 M0, n(%)**	2 (6.7)	1 (5.6)
**T3N2MX, n(%)**	2 (6.7)	1 (5.6)
**T3N3M0, n(%)**	1 (3.3)	2 (11.1)
**Missing, n(%)**	1 (3.3)	1 (11.1)
Chemotherapy Regimen		
** Carbotaxol, n(%)**	22 (73.3)	10 (55.6)
** Cisplatin + 5-FU, n(%)**	6 (20.0)	6 (33.3)
** 5FU, n(%)**	1 (3.3)	1 (5.6)
** FOLFOX, n(%)**	1 (3.3)	1 (5.6)

We obtained and completed processing all 30 subjects’ biopsy samples for BDI analysis within 24 to 72 hours. BDI analysis was successfully performed on 28/29 eligible patients’ samples, reflecting a 96.6% success rate. The single failure was due to one sample lacking sufficient tissue for complete BDI analysis. Of the 29 patients on-study, we observed no study procedure or biopsy-related complications.

### Biodynamic imaging prediction correlates with pathological response to platinum-based chemotherapy

**Table 4** presents a detailed comparison of BDI predictions to actual pathological responses. BDI predictions using a three-class neural network classifier (Grade 0, Grade 1, and combined Grades 2 & 3) aligned with clinical outcomes in 72.2% (13/18 patients) of cases (noting that random odds are 33.3%). BDI correctly identified all 6 patients with a Complete response (grade 0). For the 6 patients with a marked pathological response (Grade 1), BDI misclassified 3 cases, overpredicting in 1 and underpredicting in 2 cases. Among the 6 patients with limited to no pathological response (Grade 2 and Grade 3), BDI mis-predicted by overestimating response to treatment in 2 cases.

**Table 4:** BDI response prediction compared to patients’ pathological (clinical) response.

	Actual Pathological Response		
BDI Pathological Response Prediction	Grade 0	Grade 1	Grade 2/3
**Grade 0**	6	1	1
**Grade 1**	0	3	1
**Grade 2/3**	0	2	4

Overall, BDI showed a moderate correlation with actual clinical outcomes. For complete responses, the prediction accuracy was 100% (6 out of 6). For marked responses, the accuracy was 50% (3 out of 6). For non-responders, the accuracy was 66.6% (4 out of 6). The sensitivity for complete pathological response was 100% (6 out of 6). Its specificity was 83.3% (10 out of 12), positive predictive value (PPV) 75% (6 out of 8), and negative predictive value (NPV) was 100% (10 out of 10). It incorrectly predicted 25% (2 of 8 cases) as having a complete pathological response when they did not.

**Figure 4:**
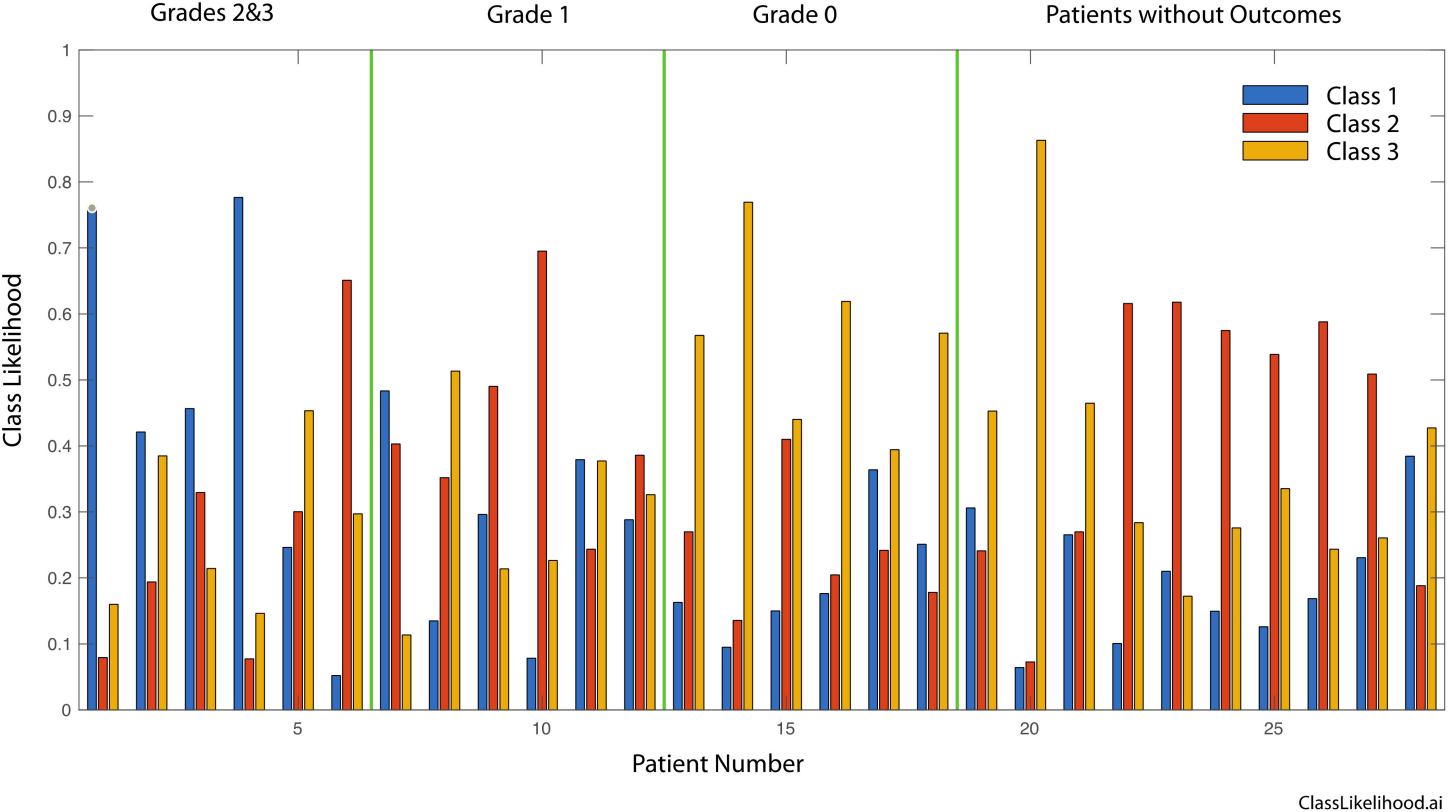
Three-class likelihoods for graded patients as well as for the patients without outcomes. Grades 2 and 3 are grouped into a single class. Respectively, Grade 0 signifies tissue sensitivity to chemotherapy and Complete Response clinically, Grade 1 signifies a mixed response to chemotherapy and a Marked Response clinically, finally Grade 2 or 3 signify Partial/No Response to chemotherapy as in the tissue is resistant to chemotherapy and tumor regression is not expect clinically.

**Figure 4** shows the likelihood for belonging to one of the three classes for all patients, grouped according to the pathological response (Grades 2&3, Grade 1 and Grade 0). The dominant likelihood is taken as the patient prediction for correlation to clinical outcomes, as in **Table 4**.

**Figure 5:**
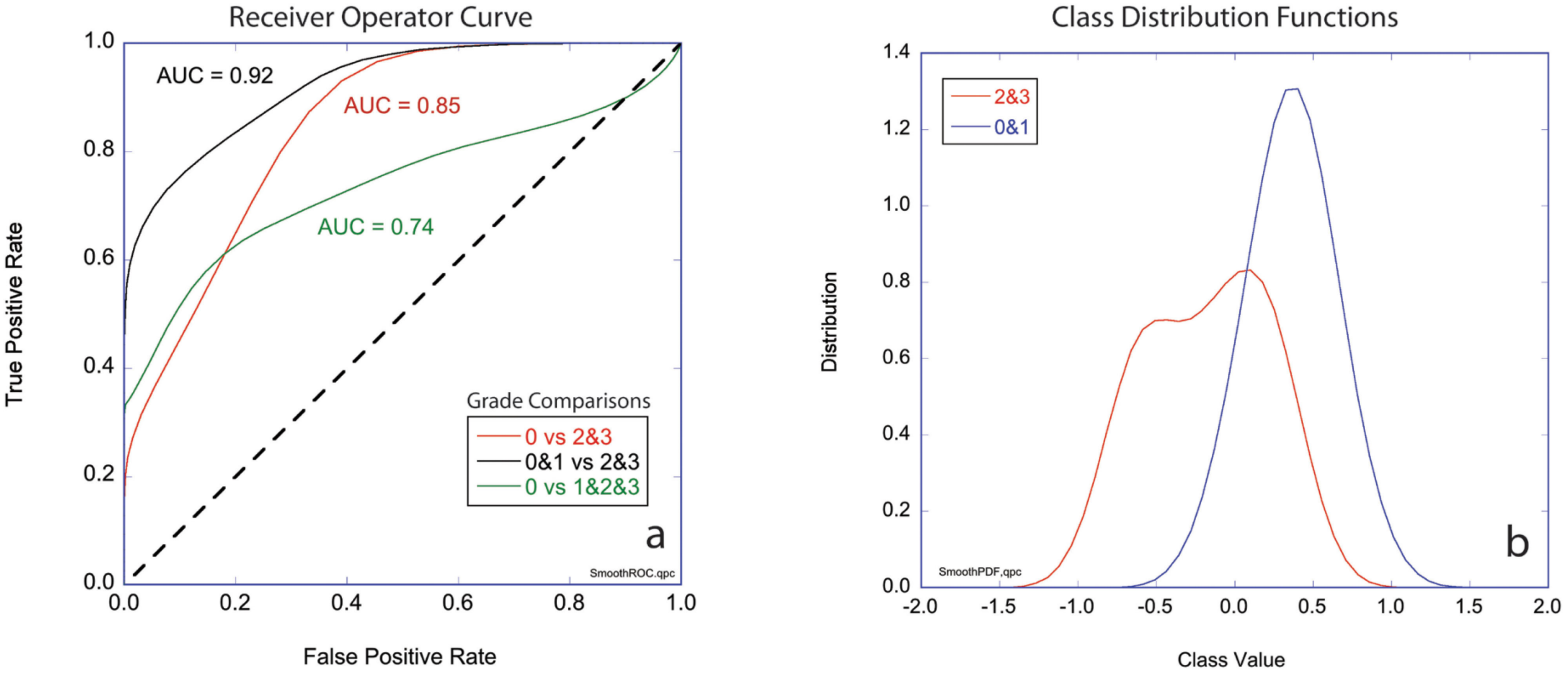
Two-class performance. **(A)** Receiver operator curves (ROCs) plotting true positive rate against false positive rate for three cases comparing groups of grades (0 vs 2&3, 0&1 vs 2&3, and 0 vs 1&2&3) with the respective values for areas under the curve (AUC). **(B)** Class distribution functions for Grade 0&1 versus Grades 2&3 (black curve in part a). The horizontal axis is the mean value of the likelihoods for non-response (-1) and response (+1). Respectively, Grade 0 signifies tissue sensitivity to chemotherapy and Complete Response clinically, Grade 1 signifies a mixed response to chemotherapy and a Marked Response clinically, finally Grade 2 or 3 signify Partial/No Response to chemotherapy as in the tissue is resistant to chemotherapy and tumor regression is not expect clinically.

The three-class likelihoods can be combined into two-class classifiers to generate receiver operator curves (ROCs) for several combinations. The ROCs for these comparisons plot true positive rate against false positive rate and are shown in **Figure 5A** for three cases: Grades 2&3 versus Grade 0, Grades 1&2&3 versus Grade 0, and Grades 2&3 versus Grades 0&1. The first case ignores the marked (but not complete) responders (AUC = 85%). The second case compares Grade 0 (complete pathological response) against all others (AUC = 92%). The third case compares the non-responders to the complete and marked responders (AUC = 74%). The diagonal in the figure represents 50/50 likelihood for a random relationship. The area under the curve (AUC) is a measure of assay reliability with a maximum 1.00 for a “perfect” assay and 0.50 for a random relationship. **Figure 5B** shows the probability distribution functions for the first case that compares the least responsive to the most responsive patients for which the AUC = 85%.

## Discussion

The application of BDI provides evidence of the feasibility of its implementation in a clinical setting. All samples were successfully collected, transported, and processed within hours, confirming BDI’s suitability for routine clinical use. We also obtained BDI results for 28 of the 29 examined samples without any biopsy-related complications.

Crucially, BDI’s preclinically observed predictive capability appears to translate to the human model of EA. Our study demonstrated that BDI predicts chemotherapy responses with 72.2% accuracy for a three-class classifier (33% random performance), a moderate yet promising success rate. This rate is comparable to the 84% accuracy for the two-class classifier (50% random performance) noted in preclinical trials (31). It is important to acknowledge the complex and heterogeneous nature of human tumors and the influence of host factors on drug responses. These factors may not have been fully captured in preclinical models, as they were trained on a limited number of canine rather than human samples.

Additionally, the underprediction in 2 cases may be attributed to the synergistic effect of radiotherapy in clinical practice, which has been shown to improve local-regional control of esophageal cancers, helping achieve a complete pathological response; however, the radiation effect was not replicated for the BDI analysis (40). It must be kept in mind that accuracy may improve with broader BDI implementation. Utilizing larger human datasets and markers would help refine biomarker selection prior to training the neural network, reducing training epochs and the size of the hidden neuron layers to decrease the possibility of overfitting.

Notably, BDI had a 100% accuracy in predicting all 6 patients with clinical complete response (grade 0). Nonetheless, it mis predicted 2 other patients as achieving grade 0. With its high sensitivity (100%), specificity (83.3%), and 100% negative predictive value, BDI has the potential to identify the subset of patients less likely to have a complete pathological response to treatment. The BDI technique shows promising implications for personalized cancer treatment. Theoretically, BDI could enable clinicians to predict tumor responses to chemotherapy prior to treatment by identifying those unlikely to respond to chemotherapy. It may also permit physicians to compare the potential impact of different regimens in tumors without cross-resistance to the available regimens, subsequently choosing the ideal treatment. Tailoring the most effective individual regimen would improve patient outcomes and minimize unnecessary toxicities.

### BDI’s advantages over other predictive tools

A key BDI strength is providing real-time tumor response data before chemotherapy initiation. These data were obtained rapidly within 24-72 hours from a tumor biopsy, avoiding any treatment delays compared to gene expression profiling methods that can take weeks.

Although BDI requires a fresh biopsy, its ability to functionally assess living tumor tissue within its native 3D microenvironment promises more accurate predictions than solely genomic, proteomic, or even 2D culture methods. Unlike surface imaging techniques, BDI can probe into tissues, providing a comprehensive view of tumor dynamics in naturally hypoxic conditions far from tissue surfaces. Distinguishing itself from tools that merely quantify static gene or protein levels, BDI offers dynamic insights into functional tumor responses like intracellular motions and viability after drug exposure. It captures crucial phenotypic information beyond genomics, such as accurate drug delivery and response kinetics and provides superior biological context. The large number of extracted biodynamic features allows for comprehensive analysis capturing tumor heterogeneity, hence predicting the regimen with the best potential response for most tumor volume. BDI bridges the best attributes of *in vitro* sensitivity assays and modern Artificial Intelligence (AI) and Machine Learning (ML) Models while sidestepping their limitations. The success of AI and ML models depends on the quality and quantity of the training data. These models can be “black boxes,” making it difficult to interpret their predictions. BDI circumvents these issues by generating a large amount of high-quality data and translating it into specific, reliable, and easily interpreted response fingerprints and outcomes.

Among the strengths of this study is the novelty of BDI technology, which offers real-time insights into chemotherapeutic response. The high rate of successful biopsy sample collection and processing further contributes to the reliability of the results. Nonetheless, several limitations warrant discussion, such as the small number of patients who underwent surgery, limiting the training set for the neural network. The pilot nature of this study limits the generalizability of our findings. The study does not account for the effects of radiotherapy, a standard treatment for locoregional EA received by all participants, that is synergistic with chemotherapy contributing to the pathological response. Furthermore, the classification of drug response derived from previous preclinical work may only partially translate to human tumors. Finally, the study population, while reflecting the sex demographics of esophageal adenocarcinoma patients, lacked diversity, being predominantly white, limiting generalizability across other populations. Further large-scale blinded studies in diverse cohorts are needed to substantiate BDI’s predictive accuracy before clinical implementation. The inherent spatial heterogeneity of tumors and the potential non-representativeness of a single fine needle biopsy sample may explain some discrepancies between BDI’s predictions and clinical outcomes and must be addressed in subsequent research.

## Conclusion

Biodynamic Imaging has demonstrated high feasibility for clinical application and promising efficacy in predicting chemotherapy response in locoregional esophageal adenocarcinoma, especially among poor responders. The technology’s rapid-response capability and maintenance of the biopsy’s 3D architecture are key strengths supporting its potential use in personalized treatment strategies. Integrating BDI with other predictive modalities could yield a more robust and multidimensional approach to treatment planning. Exploring the applicability of BDI to other malignancies and treatment modalities, such as radiotherapy and immunotherapy, could further establish its versatility as a predictive tool. After additional validation, BDI may guide treatment prospectively when deciding between different regimens. Furthermore, with its predictive ability, BDI may hold promise as a tool to test the development and efficacy of new cancer treatments.

## Data Availability

The raw data supporting the conclusions of this article will be made available by the authors, without undue reservation.

## Appendix A

**Table:** BDI predicted response to chemotherapy and clinical pathological response to chemotherapy after surgery by subject.

Study ID	BDI Grade 0 probability	BDI Grade 1 probability	BDI Grades 2 or 3 probability	Pathological Grade from BDI	Clinical pathological Grade from surgery, collapsing Grade 2 and 3
**2**	0.567	0.270	0.163	Grade 0	Grade 0
**4**	0.513	0.352	0.135	Grade 0	Grade 1
**7**	0.214	0.490	0.296	Grade 1	Grade 1
**9**	0.214	0.329	0.457	Grade 2/3	Grade 2/3
**11**	0.769	0.136	0.095	Grade 0	Grade 0
**12**	0.146	0.077	0.777	Grade 2/3	Grade 2/3
**13**	0.453	0.300	0.246	Grade 0	Grade 2/3
**14**	0.440	0.410	0.150	Grade 0	Grade 0
**18**	0.227	0.695	0.078	Grade 1	Grade 1
**19**	0.619	0.205	0.176	Grade 0	Grade 0
**20**	0.160	0.079	0.761	Grade 2/3	Grade 2/3
**22**	0.394	0.242	0.364	Grade 0	Grade 0
**24**	0.385	0.194	0.421	Grade 2/3	Grade 2/3
**26**	0.571	0.178	0.251	Grade 0	Grade 0
**29**	0.377	0.244	0.379	Grade 2/3	Grade 1
**32**	0.326	0.386	0.288	Grade 1	Grade 1
**33**	0.114	0.403	0.483	Grade 2/3	Grade 1
**34**	0.297	0.651	0.052	Grade 1	Grade 2/3
